# Middle school students’ mathematical problem-solving ability and the influencing factors in mainland China

**DOI:** 10.3389/fpsyg.2022.1042315

**Published:** 2022-11-11

**Authors:** Zhuzhu Xu, Chunxia Qi

**Affiliations:** ^1^School of Education, Shanghai International Studies University, Shanghai, China; ^2^Faculty of Education, Beijing Normal University, Beijing, China

**Keywords:** influencing factors, mathematical problem-solving, middle school mathematics, mainland China, benchmark, proficiency

## Abstract

This study investigated the mathematical problem-solving ability of 42,644 ninth-grade students who participated in regional education quality health monitoring from Z province in East China and the factors which influence their performance of mathematical problem-solving. The results are as follows: (1) ~96% of the students’ mathematics problem-solving ability meets the basic academic requirements of the mathematics curriculum standards; (2) boys and children without siblings performed better, and urban students performed significantly better than county and rural students; (3) ~28% of students’ mathematical problem-solving performance came from inter-school variability; urban and rural backgrounds had a greater impact on mathematical problem-solving than did teaching factors, while teaching self-efficacy had the least impact among the school-level influencing factors. In contrast, the influence of individual non-intelligence factors was higher than that of student background variables, including a greater positive effect of self-efficacy and a greater negative effect of mathematics anxiety.

## Introduction

Problems inspire the search for knowledge and learning. As such, Zhang (2012) suggests that personal learning and knowledge acquisition are pursued to solve practical difficulties. Thus, the purpose of mathematics learning is to solve various problems in the mathematical context (Ma, 2009). The role of science is not only to explain the different phenomena in the world, but also to solve real-world problems. Thus, problems drive scientific development. Historically, mathematical science developed from two cultural traditions and two models. Culturally, mathematics is derived from Western abstract deductive mathematics represented by ancient Greek mathematics and algorithmic applied mathematics represented by ancient Chinese mathematics (Liu, 2005). The confluence of these two traditions, neither of which can be solved without mathematical constructs, formed modern mathematics. Polya (1944) argued that one of the main purposes of mathematics education is to develop students’ problem-solving ability and teach students how to think. The indispensable role of the ability to solve problems using mathematics in the process of mathematical exploration, discovery, and innovation has gradually attracted widespread global attention. Mathematical problem-solving ability has been introduced into global curriculum reforms (National Council of Teachers of Mathematics (NCTM), 1980; Ministry of Education of the People’s Republic of China, 2012; Wang, 2021) and international evaluations (OECD, 2013). In addition, scholars from the East and West have focused on the important factors that influence students’ performance of mathematical problem-solving. They can be broadly summarized as internal factors of the individual learner (e.g., cognitive resources, meta-cognition and non-intellectual factors), external factors (e.g., complexity, familiarity, type, context of the problem), and teaching factors (Schoenfeld, 1985; Mayer, 1992). But generally speaking, at present, the academic community have not paid enough attention to the non-intellectual factors and teaching methods (Wang, 2000). Moreover, comparisons reveal that American mathematics education promotes the development of students’ mathematical literacy or other core abilities, wherein the problem-solving process focuses on the application of mathematics knowledge and skills. In contrast, Chinese mathematics education has long advocated *double-base teaching*, which promotes a process of mathematical problem-solving that focuses on the acquisition of basic knowledge and skills instead of reasoning activities (Peng et al., 2017). While numerous studies suggest that Chinese and East Asian students’ overall math problem-solving skills surpass those of Western students, such as those in the United States, various studies indicate that no significant gap exists between the two in solving complex mathematical problems (Zhao and Shen, 2003). In fact, the higher mathematics achievement of middle school students in mainland China is inextricably linked with the learning process of mathematical problem solving. In particular, China’s compulsory education middle school mathematics curriculum standard also emphasizes that students should cultivate mathematical affections in mathematics learning and actively exert the important promotion of non-intelligence factors (Ministry of Education of the People’s Republic of China, 2012). Thus, under the advocacy of domestic *double-base teaching*, how do Chinese students develop their mathematical problem-solving ability? Which factors have a greater impact on it? These questions still urgently require an intensive investigation of the overall mathematical problem-solving process of mainland Chinese students, especially to determine the key internal and external factors that influence their mathematical problem-solving performance.

## Literature review

### Significance and value as goals of mathematics teaching

The advantage of improving problem-solving ability as a goal of mathematics teaching has long been recognized. Since the 1980s, most countries have regarded improving students’ problem-solving ability as one of the primary goals of mathematics teaching (Silver and Kilpatrick, 1988; Kilpatrick, 2009). For instance, in 1980, the National Council of Teachers of Mathematics (NCTM) proposed establishing problem-solving as the core of the mathematics curriculum, thereby introducing a primary goal of American mathematics education [National Council of Teachers of Mathematics (NCTM), 1980]. In 1982, the United Kingdom stated that the core of mathematics education is to cultivate the ability to solve mathematical problems, emphasizing that mathematics is meaningful only when it is applied to various situations [Department for Education and Science (DES), 1982]. Since then, many countries have addressed this issue. In 1989, Japan formally integrated the content of *Subject-Studying*, based on a mathematics class featuring problem-solving, in its newly revised *Curriculum Guidelines* (Fang et al., 1993). In 1990, Singapore’s mathematics syllabus listed the development of students’ mathematical problem-solving ability as the basic goal of the mathematics curriculum and, for the first time, proposed the pentagonal model of the mathematics curriculum framework, with mathematical problem-solving positioned as its core (Fan and Zhu, 2003). Currently, most countries regard improving students’ problem-solving ability as an important goal of mathematics education, and problem-solving has become a popular topic in international mathematics curriculum and teaching research (Stacey, 2005; Manfreda, 2021).

In contrast, the People’s Republic of China (1949–1957) was influenced by the educational climate of the time and adopted the Soviet mathematics teaching model, which emphasized abstraction, rigor, and application. It was not until the late 1970s that elementary and secondary mathematics syllabi noted that students should learn to apply mathematics knowledge to solve real-world problems. In modern China, the *Mathematics Curriculum Standards for Compulsory Education* (2011) consider problem-solving as the basic goal of school mathematics (Ministry of Education of the People’s Republic of China, 2012), including the later *Ordinary High School Mathematics Curriculum Standards* (2002) and *General High School Mathematics Curriculum Standards* (2017). These curriculum standards emphasize learning to discover and pose problems from the perspective of mathematics, apply mathematics knowledge to solve practical problems, enhance application awareness, and improve practical ability (Ministry of Education of the People’s Republic of China, 2003, 2020). As such, although China’s research on problem-solving began relatively late, it has developed rapidly and is generally valued by the domestic mathematics education community.

### Mathematical problems and problem-solving

American mathematician Halmos (1995) argues that the fundamental element of mathematics is the problem and answer, and the problem is the heart of mathematics. Thus, scholars from various countries have investigated what constitutes the problem. Polya (1965) states that a mathematical problem means to drive learners to find appropriate actions to achieve a visible—but not immediately accessible—goal. Similarly, several Japanese scholars believe that problem situations refer to those that do not yet have a direct solution, thus resulting in a cognitive challenge situation (Chen, 2007). Moreover, according to a renowned Chinese mathematics educator, a mathematics problem is a situation that a person wishes to comprehend, but for which standard solutions cannot be applied (Zhang, 1991). Therefore, mathematical problems refer to problems that learners can only solve through active exploration and thinking using existing mathematical concepts, theories, or methods.

However, consensus has also not yet been achieved regarding the concept of mathematical problem-solving. Perspectives can generally be classified into five categories: (1) mathematical problem-solving refers to facing new situations and issues in daily life and social practice that contradict subjective and objective needs and have no ready-made countermeasures, requiring psychological activity to seek solutions to problems that occur (Shao, 1983; Zhao, 2007); (2) mathematical problem-solving is considered to be the process of applying previously learned knowledge to new and unfamiliar situations (Tan, 2004); (3) mathematical problem-solving, as an important part of curriculum theory, is a type of teaching (Dai, 2012); (4) problem-solving is perceived as the purpose of mathematics teaching (Department for Education and Science (DES), 1982; Pasani, 2018); and (5) mathematical problem-solving is defined as the ability to apply mathematics to various situations (Mayer, 1992; Stacey, 2005). Despite the apparent inconsistency in the formation of problem-solving, the preceding explanations emphasize that mathematical problem-solving is not only an essential skill for all students, but also a process in which they use a variety of intellectual activities to find solutions to problems. In addition, it requires teachers to provide students with an environment and opportunities for discovery and innovation in the classroom. Furthermore, for students, mathematical problem-solving refers to the comprehensive and creative application of mathematical knowledge and methods to solve problems that are not pure exercises, including practical problems and problems derived from mathematics.

### Psychological analysis of the process of mathematical problem-solving

Mathematical problem-solving is not only the core of mathematics education but also an important part of mathematics learning psychology. Therefore, research on the psychological mechanism of problem-solving is intriguing. However, various psychological theories maintain different interpretations of problem-solving, and there is no comprehensive view to date. The previous behaviorist theory considered problem-solving to be trial and error, while the Gestalt theory considers it to involve insight (Kilpatrick, 1978; Lumbelli, 2018). Actually, in the process of problem-solving, trial and error and insight are not mutually exclusive and often occur alternately. In addition, depending on its nature, a problem can be solved through trial and error or by relying on insight. Moreover, these behaviors are not entirely random but are organized behaviors that gradually search for information, establish connections between information, and adopt certain strategies. Cognitive psychology, a prevalent approach in Western psychology (Neisser, 1967), has largely promoted the theory of mathematics education. The information processing theory developed from cognitive psychology states that problem-solving is a process of finding, receiving, and processing information (Newell and Simon, 1972; Chien et al., 2016).

Based on psychological analyses of the process of solving mathematical problems, more researchers began to focus on the steps and procedures of problem-solving, especially observing the process of solving complex mathematical problems (Duncker, 1945; Hunt, 1968). The theory of information processing gradually aroused people’s interest in the role of heuristic methods in the problem-solving process. The most influential was Polya’s (1957) four-stage problem-solving process: understanding the problem, devising a plan, implementing the plan, and reviewing and testing. In addition, Mayer et al. (1991) also categorize the problem-solving process into three stages: paraphrasing, integration, and planning. In recent years, an increasing number of related studies on problem-solving steps and procedures, such as heuristic training (Wang, 2020), discovery learning (Hulukati et al., 2018), and other teaching procedures (Goulet-Lyle et al., 2019) have been applied to the teaching field.

### Influencing factors of mathematical problem-solving

Factors that affect the solution of mathematical problems are elements that impact the problem-solving process. As problem-solving is a complex psychological process, it requires students to process the conditions, reorganize known concepts and theorems from the understanding of the basic relationship and characteristics of the problem, adjust the relationship between the basic elements in the problem, and explore and guess problem-solving strategies and methods. Based on the extant literature, many factors—such as knowledge, experience, motivation, confidence, thinking ability, and meta-cognition (Wang, 2017)—influence mathematical problem-solving. These factors can be classified into three categories: (1) the learner’s individual internal factors, such as personal experience (personal characteristics of the problem solver), cognitive factors (intuition, imagination, abstraction, generalization, reasoning, analysis, and synthesis), meta-cognition, and non-intellectual factors, such as care, desire, motivation, interest, will, and belief (Ye and Zhang, 2004; Tan, 2009); (2) external factors related to the mathematical problem, such as complexity, familiarity, type, and context of the problem (OECD, 2013); and (3) teachers’ problem-solving teaching, such as teaching self-efficacy of problem-solving and teaching methods for problem-solving (Schoenfeld, 1985).

### Evaluation of mathematical problem solving ability at home and abroad

Although many scholars have conducted in-depth research on the steps, procedures, and open-ended questions of mathematical problem-solving, no unified and clear framework and standard for evaluating the ability of mathematical problem-solving exists. For instance, Mayer et al. (1991) designed 18 arithmetic problems using their original problem-solving procedures based on their previous psychological analysis of the mathematical problem-solving process, and the problems were used to compare the performance of English and Japanese fifth-grade students in mathematical problem-solving. However, mathematical problem-solving is not a single component, but an ability that involves simple calculations and reading comprehension as well as extensive reasoning skills (Kilpatrick, 1978). Various Chinese scholars believe that junior high school students’ mathematical problem-solving abilities involve the four major ability elements of reading comprehension, mathematical modeling, problem-solving expression, and evaluation reflection (Bai, 2011). Thus, the mathematical problem-solving evaluation tools developed by scholars have gradually transitioned from simple to complex, and the problem form has changed from closed to open. As such, early mathematical problem-solving tests usually focused on the preparation of traditional arithmetic problems (Stinger et al., 1990). Later, various researchers began to design high-level cognitive diagnostic tools, such as the QUSAR Cognitive Assessment Instrument (QCAI), which highlights the important role of open-ended questions in mathematical problem-solving (Lane, 1993). On this basis, various studies have applied these open-ended problems related to cognitive diagnostic tools to specific problem-solving evaluations. Cai (1995) used the QCAI as a test tool in a comparative study on the mathematical problem-solving ability of sixth-grade students in China and the United States. Ding et al. (2009) also used the QUSAR QCAI in their study of the relationship between the elementary school mathematics classroom environment and students’ problem-solving ability; they concluded that the dimensions of “happy” and “knowledge-related” in the classroom environment scale had a significant positive predictive effect on students’ problem-solving ability and traditional test scores. All in all, few studies have examined the measurement and evaluation of mathematical problem-solving processes or comprehensively considered the relevant influencing factors of the mathematical problem-solving process. Evaluation design concepts are only incorporated in some representative mathematics curriculum standards and the evaluation framework of international comparison projects (Xu and Qi, 2018).

## Analysis framework

On the whole, compared with foreign research on mathematical problem-solving, Chinese mathematics education pays special attention to the learning of mathematical problem-solving strategies and skills, such as in-depth analysis of external factors like the form, background and other elements of mathematical problems, but little attention is paid to the analysis of students’ internal cognitive process of mathematical problem solving. On the other hand, although the domestic mathematics curriculum standards for middle schools also emphasize the role of non-intellectual factors such as mathematical affections in promoting learning, their attention is still obviously insufficient in the actual evaluation (Wang, 2000). In fact, research suggests that personal internal psychological factors, such as motivation, learning interest, and self-efficacy, are more significant in mathematical problem solving performance (Sun et al., 2016). Moreover, the impact of teaching factors on students’ mathematical problem-solving performance also cannot be ignored (Schoenfeld, 1985). Therefore, to systematically evaluate the mathematical problem-solving ability, in addition to considering examining the structural elements of mathematical problem solving, the role of internal non-intellectual factors and teaching variables in the process of problem-solving must be valued.

In addition, the empirical investigation on the influencing factors of mathematical problem solving in the existing research is more just for the perspective of students or only considering the intervention of the teaching environment, so it is rare to combine these two together for comprehensive analysis. Therefore, at the technical level, multilevel models can be used to analyze the predictive effect of influencing factors at different levels (such as student level and school level) on the performance of middle school students’ mathematical problem-solving ability, thus helping to find the key influencing factors in the school education environment, so as to promote the cultivation and improvement of students’ mathematical problem-solving ability ultimately.

As such, on the basis of implementing academic requirements in the *Chinese Compulsory Education Mathematics Curriculum Standards*, this study designed test papers for evaluating students’ mathematical problem-solving ability and questionnaires focusing on non-intellectual internal factors and teaching variables which affect students’ mathematical problem-solving performance. It is hoped that this research can help the academic community to clearly clarify the current performance of middle school students’ mathematical problem solving in mainland China, as well as the learning differences between student groups, schools and regions, and find the key factors that restrict the cultivation of students’ mathematical problem solving ability, so as to provide targeted strategies for improving mathematical problem solving ability. The following research questions were posed:

1. What is the overall proficiency of middle school students’ mathematical problem-solving ability in mainland China?
2. Do middle school students’ mathematical problem-solving ability differ based upon gender and urban–rural environment?
3. What are the key factors that influence middle school students’ mathematical problem-solving performance?

## Materials and methods

### Participants

This study utilized 2016 survey data provided by the *Regional Education Quality and Health Monitoring* team of the China Basic Education Quality Monitoring Collaborative Innovation Center. The *Regional Educational Quality and Health Monitoring* project is an important regional education investigation and evaluation program in China that is implemented annually. The program aims to conduct health monitoring on the quality of domestic mathematics education through standardized tests and questionnaires based on Chinese mathematics curriculum standards, and it proposes targeted improvements based on data analysis and evaluation. This study adopted a three-stage unequal probability sampling method. The first stage utilized the stratified probability proportionate to size (PPS) sampling method to extract counties (cities and districts). The second stage applied the hierarchical PPS method to extract schools. The third stage used random equidistant sampling to select students. Consequently, the sampling results provided a sample that was representative of the overall province and distribution of different groups, including cities, counties, towns, and rural areas. The study selected 42,968 ninth-grade students, who participated in the 2016 *Regional Education Quality and Health Monitoring* (used as the main data source), from 762 schools of Z province in East China. In addition, in terms of imputation, since the sample is large enough and the missing rate is only 0.75%, this study used the method of list-wise deletion to obtain 42,644 valid samples, including 22,302 boys (52.3%) and 20,342 girls (47.7%).

### Instruments

This study was based on the *Regional Education Quality and Health Monitoring* project, which included middle school students’ mathematical problem-solving test papers and student and teacher questionnaires on the factors influencing mathematical problem-solving.

#### Mathematical problem-solving test paper

The middle school mathematical problem-solving assessment of the *Regional Education Quality and Health Monitoring* project was guided by the *Mathematics Curriculum Standards for Compulsory Education* (2011), drawing on the experience of large-scale international mathematics assessment, this study designed the test paper for evaluating three dimensions (content, context and cognitive) of mathematical problem-solving process and questionnaires focusing on non-intellectual internal factors and teaching variables that affect students’ problem-solving performance. In this study, mathematical problem solving was defined as an individual’s ability to use cognitive processes to face and solve real, interdisciplinary problems. The mathematical problem-solving test paper consists of 10 items, including numbers and algebra, figures and geometry, and statistics and probability as the content dimensions to examine mathematical problem-solving ability; these items also involve three contexts: personal situation, social situation, and pure mathematical situation. Meanwhile, the cognitive processes involved in problem-solving are divided into three domains: knowing (four items), understanding (four items), and applying (two items), respectively. The test paper contains multiple-choice questions and subjective questions (including open-ended questions), with items including two to three questions. The difficulty of the test paper is about 0.70, the discrimination ranges between 0.40 and 0.80, about 76% of the items’ discrimination is >0.40, and the internal consistency of the test paper is >0.9, which indicates that its reliability is good.

#### Questionnaires on factors influencing the performance of mathematical problem-solving

Based on the extant literature, a questionnaire was designed to identify factors affecting the performance of middle school students in solving mathematics problems. The significance of related influencing factors was investigated from the perspectives of students and teachers. Two questionnaires were compiled—one for students and another for teachers. The student questionnaire included four subscales: mathematics anxiety, mathematics interest, self-efficacy, and teacher–student relationship. The three subscales of mathematics anxiety, mathematics interest, and self-efficacy were adapted from the *Student Questionnaire* in PISA (translated into the Chinese version scales by the research team for application). Answers were given on a 5-point Likert scale ranging from 1 (*strongly disagree*) to 5 (*strongly agree*). Higher scores indicated higher degrees of expression. Teacher–student relationship comprised a self-reported subscale rated on a 5-point Likert scale ranging from 1 (*strongly disagree*) to 5 (*strongly agree*). The higher the score, the more harmonious the teacher–student relationship. In addition, to focus on the impact of teaching factors on students’ mathematical problem-solving, the teaching self-efficacy of problem-solving and teaching methods for problem-solving subscales, rated on a 5-point Likert scale, were added to the teacher questionnaires for middle school mathematical problem-solving monitoring. Moreover, the internal consistency coefficients of the overall student questionnaire and teacher questionnaire were 0.91 and 0.89, respectively, and both types of questionnaires had good structural validity (CFI = 0.910, RMSEA = 0.054; CFI = 0.921, RMSEA = 0.070).

### Data collection and test procedure

In order to collect test data quickly and efficiently, the China Basic Education Quality Monitoring Collaborative Innovation Center cooperated with the Department of Education in Z Province to jointly launch the project of *Regional Education Quality and Health Monitoring*. With the assistance of cities and counties (county-level cities and districts) in Z province, sampling tests were successfully organized and implemented in 11 cities and 104 districts and counties in Z Province in October 2016. Among them, 42,968 ninth grade students from 762 junior high schools participated in this test, while 42,644 of them finally filled out the *Student Questionnaire*. In addition, a total of 3,565 principals and vice-principals in charge of teaching of the participating schools filled out the *Principal Questionnaire*, 10,599 teachers answered the *Teacher Questionnaire*, and a total of 76,502 parents of students answered the *Parent Questionnaire*.

Meanwhile, for the test procedure, the mathematics project team has undergone a series of complete evaluation processes from the beginning of 2016 to November 2016, including framework testing and two-way specification table preparation, item collection and polishing, the first interviews with six participants, the round pre-tests of 30 participants, the second-round pre-tests of 300 participants, and the external reviews of domestic and foreign mathematics experts and assessment experts. Thus, implementing these procedures ultimately ensures the scientific and normative nature of the entire testing process (Qi et al., 2015).

### Data processing

After going through the above test procedures, the project team first determined the scoring standards based on the standard answers of the test paper of mathematical problem-solving and the students’ final formal participation in the test and then scored objectively according to the scoring standards. Next, the Rasch model from item response theory was used to analyze students’ original scores to obtain their mathematical problem-solving ability value. Then, the ability value was converted into a standardized score (average 300, standard deviation 50), that is, the scale score that represents students’ mathematical problem-solving performance. Simultaneously, the project team used the Angoff method^1^ to calibrate the performance of students’ mathematical problem-solving ability, and it divided the students into four levels (A, B, C, D) according to their mathematical problem-solving performance, where level C represents the benchmark of students’ mathematical problem-solving. In contrast, the project also processed the original data from student and teacher questionnaires into a questionnaire database. In addition, this study first used the descriptive statistical analysis method to further describe the proficiency of students’ mathematical problem-solving; then, it used the hierarchical linear model to analyze the inter-school differences in mathematical problem-solving performance and the predictive role of factors from different educational levels.

## Results

### Overall proficiency of students’ mathematical problem-solving ability

To distinguish the characteristics of mathematical problems of different difficulty levels and the characteristics of students’ mathematical problem-solving performance, this study divides the performance of all students’ mathematical problem-solving ability into three proficiency levels from high to low, namely A level, B level, and C level, with each level representing the expected range of abilities for a different student group. Among them, students at the A level can comprehensively use basic knowledge in the process of mathematical problem-solving, master mathematical concepts, apply appropriate mathematical methods, or establish appropriate mathematical models to solve unfamiliar or open-ended problems. The group of students at the B level can understand the characteristics of mathematical concepts in the mathematical problem-solving process and apply appropriate mathematical methods or build simple mathematical models to solve relatively unfamiliar or unpracticed problems. Finally, students at the C level can only memorize and identify mathematical concepts in the mathematical problem-solving process and use conventional mathematical methods to solve familiar or practiced problems. In addition, below C level is defined as D level; the students at this level cannot analyze and interpret the answers nor evaluate and categorize problem-solving processes and methods. Table 1 shows that the mathematics problem-solving ability of middle school students in Z province in mainland China is relatively good; the majority of students’ mathematical problem-solving skills are at a moderate to high level, and 48% of them have reached the A level, 35% the B level, and 13% the C level, with only 4% having located in the D level.

**Table 1:** The ratio of different proficiency levels of students’ mathematical problem-solving ability.

Students’ mathematical problem-solving proficiency in mainland China	Proficiency levels			
	A level	B level	C level	D level
Ratio	48%	35%	13%	4%

### Background differences in the benchmark of students’ mathematical problem-solving performance

As mentioned above, the C level represents the benchmark for students’ mathematical problem-solving performance^2^, which is the minimum requirement for middle school students’ problem-solving skills in the *Mathematics Curriculum Standards for Compulsory Education* (2011). In other words, when a student’s mathematical problem-solving proficiency reaches the C level and above, their problem-solving ability meets the curriculum standard’s academic requirements. The survey found that 98% of boys’ mathematics problem-solving ability reached the C level and above, 3 percentage points higher than girls, and the gender difference was significant (*p* < 0.01, *φ* = 0.12). Simultaneously, the proportion of only children (97%) reaching the C level and above was also significantly higher than that of non-only children (94%), and we observed a significant difference between the two (*p* < 0.01, *φ* = 0.11). In contrast, we found no significant difference between leftover students and non-leftover students in the compliance rate of the benchmark of mathematical problem-solving ability (*p >* 0.05, *φ* = 0.06), but the proportion of non-leftover students (96%) reaching the C level and above was slightly higher than that of leftover students (95%). In addition, we observed significant urban and rural differences in the performance of middle school students’ mathematical problem-solving ability (*p* < 0.01, *φ* = 0.21), and 98% of urban students’ mathematical problem-solving ability reached the C level and above, which was 1 and 3 percentage points higher than that of county students and rural students, respectively (see Table 2).

**Table 2:** The compliance rate of middle school students’ mathematical problem-solving performance.

The compliance rate of middle school students’ mathematical problem-solving performance in mainland China (C level and above)			Ratio
Background variables	Gender	male	98%
		female	95%
	The only child situation	only children	97%
		non-only children	94%
	The leftover situation	leftover	95%
		non-leftover	96%
	Urban and rural background	Urban	98%
		County	97%
		Rural	95%

## Students’ mathematical problem-solving ability and influencing factor model setting

Our hierarchical linear model took students’ mathematical problem-solving ability (the scale score) as the dependent variable; gender, leftover situation, only-child situation, mathematics interest, self-efficacy, teacher–student relationship, and mathematics anxiety as the student-level variables; and urban and rural backgrounds, teaching self-efficacy of problem-solving, and teaching methods for problem-solving as the school-or teacher-level variables.

### Hierarchical linear model analysis

Due to the nested structure of the school-and student-level data, this study used the hierarchical linear model^3^ to process them. Compared with the traditional regression method, this method can make full use of the data information of each level in the analysis of differences in mathematical problem-solving performance and decompose differences at each relevant level; thus, the source and size of the difference can be estimated more accurately. The analysis process involved two basic models: the null model and the random intercept model. The following analysis shows the regression equation model and the corresponding variance component analysis results after including the student-level variables and the school-level variables, respectively (see Table 3).

**Table 3:** Students’ mathematical problem-solving performance and influencing factors HLM analysis results.

	Variables	Model 0	Model 1	Model 2
Student level variables	Gender (Male–Female)		−6.54^**^	−6.12^**^
	The only child situation (Yes–No)		−5.58^**^	−4.79^**^
	The leftover situation (Yes–No)		2.98	0.37
	Math interest			10.06^**^
	Self-efficacy			15.34^**^
	Teacher-student relationship			3.66^**^
	Math anxiety			−14.08^**^
School level variables	Urban and rural background (Urban-County-Rural)		−18.11^**^	−14.91^**^
	Teaching self-efficacy of problem solving			1.51^*^
	Teaching methods to problem solving			4.63^**^
Variance estimation	Student level	4245.00	4236.40	3438.87
	School level	1670.21	1389.61	1013.48

The variance estimation results are nonstandard residual estimates, both of which are significant when *p* value is 0.001.

#### Model 0


(Level 1) $$Y_{ij}=\beta_{0j}+r_{ij}$$

(Level 2) $$\beta_{0j}=\gamma_{00}+\mu_{0j}$$

In Model 0, *Y_ij_* is the mathematical problem-solving performance of *i* students in *j* school; *β_0j_* is the average problem-solving performance of *j* school; *r_ij_* is the random error of individual students, which indicates the difference between the *i* students in *j* school and the *j* school’s average problem-solving performance; and *γ*_00_ is the overall average performance. *μ_0j_* is the school’s random error, which indicates the difference between the average problem-solving performance of the *j* school and the overall average performance.

Based on Model 0’s student level, we establish Model 1, which adds variables denoting students’ gender (male, female), only-child situation (yes, no), and leftover situation (yes, no), and school-level variables denoting urban versus rural background (urban, county, or rural), which study the influence of background variables on students’ mathematical problem-solving.

#### Model 1


$$\begin{array}{l} Y_{ij}=\beta_{0j}+\beta_{1j}\left(gender\right)+\beta_{2j}\left(only-childsituation\right)\\ +\beta_{3j}\left(leftoversituation\right)+r_{ij}; \end{array}$$

$$\beta_{0j}=\gamma_{00}+\gamma_{01}\left(urbanorruralbackground\right)+\mu_{0j};$$

$$\beta_{1j}=\gamma_{10};\beta_{2j}=\gamma_{20};\beta_{3j}=\gamma_{30}.$$

In Model 2, the following student-level variables are added: mathematics interest, self-efficacy, teacher–student relationship, and mathematics anxiety, which study the influence of individual non-intellectual variables on students’ mathematical problem-solving. Meanwhile, teaching self-efficacy of problem-solving and teaching methods for problem-solving are added into the school level to study the influence of teaching-related variables on students’ mathematical problem-solving.

#### Model 2


$$\begin{array}{l} Y_{ij}=\beta_{0j}+\beta_{1j}\left(gender\right)+\beta_{2j}\left(only-childsituation\right)\\ +\beta_{3j}\left(leftoversituation\right)+\beta_{4j}\left(mathematicsinterest\right)\\ +\beta_{5j}\left(self-efficacy\right)+\beta_{6j}\left(teacher–studentrelationship\right)\\ +\beta_{7j}\left(mathematicsanxiety\right)+r_{ij}; \end{array}$$

$$\begin{array}{l} \beta_{0j}=\gamma_{00}+\gamma_{01}\left(urbanorruralbackground\right)\\ +\gamma_{02}\left(teachingself-efficacyofproblem-solving\right)\\ +\gamma_{03}\left(teachingmethodsforproblem-solving\right)+\mu_{0j}; \end{array}$$

$$\begin{array}{l} \beta_{1j}=\gamma_{10};\beta_{2j}=\gamma_{20};\beta_{3j}=\gamma_{30};\beta_{4j}\\ \;\;\;\;\;\;\;\;\;=\gamma_{40};\beta_{5j}=\gamma_{50};\beta_{6j}=\gamma_{60};\beta_{7j}=\gamma_{70}. \end{array}$$

Model 0 represents the variance component analysis. By calculating the intraclass correlation coefficient (ICC), this study found that the ICC of the influential factors of ninth-grade students’ mathematical problem-solving performance was about 28%, indicating that 28% of the problem-solving performance differences in middle school students in China’s compulsory education come from inter-school differences. In other words, the model shows significant inter-group differences, and thus, it is necessary to use a hierarchical linear model for the analysis (Zhang et al., 2005).

After incorporating the background variables (Model 1), this study found that the student-level background variables (gender, the only-child situation, and the leftover situation) have little effect on students’ mathematical problem-solving performance. Further observation of the regression coefficients of these student background variables showed that the mathematical problem-solving performance of boys was higher than that of girls, and the mathematical problem-solving performance of only children was higher than that of non-only children. By contrast, the urban or rural background, which belonged to school-level background variables, had a larger impact on the average school achievement (the absolute value of the regression coefficient was larger); specifically, the mathematical problem-solving performance of urban students was significantly higher than that of county and rural students. The above findings also corroborate the results of the previous Chi-squared test.

By observing Model 2, we found that the variance of the student-level residuals reduced more when mathematics interest, learning self-efficacy, teacher–student relationship, and mathematics anxiety were added into the student-level variables. Among these individual non-intelligence factors, the absolute value of the regression coefficient of self-efficacy was the largest, followed by mathematics anxiety and mathematics interest, and the smallest was teacher–student relationship. Simultaneously, the addition of two variables that belonged to school-level, namely teaching self-efficacy of problem-solving and teaching methods for problem-solving, greatly reduced the residuals at the school level. Although they were not as prominent as the effect of urban or rural background on mathematical problem-solving performance, teaching self-efficacy and teaching methods for problem-solving did have a significant impact on students’ mathematical problem-solving, and teaching methods for problem-solving had a relatively larger positive effect.

## Discussion

### Overall performance of students’ mathematical problem-solving ability

This study shows that in the four-level distribution of students’ problem-solving ability performance, 96% of middle school students in Z province met the minimum requirements of the curriculum standard, and only 4% of students did not. This result is similar to the average level of problem-solving performance of students from OECD countries and regions that participated in the PISA 2012 test. For example, according to the students’ problem-solving performance in the PISA 2012 survey report, the proportion of students in OECD countries and regions whose problem-solving ability was at level 1 and above was 91.8, and 8.2% of the students were still unable to reach the problem-solving benchmark. However, the difference is that in terms of problem-solving performance at the high level of difficulty, the performance of students from Z province in mainland China is more prominent, with the proportion of students at the A level and above as high as 48%, while the proportion of East Asian students at level 5 and above who participated in the PISA problem-solving test is lower than 20%, of which Hong Kong-China is 19.3%, and Chinese Taipei and Shanghai-China are both 18.3% (OECD, 2014). The above results may be due to Z province being located in East China, where China’s education and economy are relatively developed. In fact, East China has always played an important role in the six administrative regions of mainland China, with its population and GDP accounting for more than 30% of the country. Moreover, in terms of basic education, the government of East China attaches importance to education investment, with well-equipped teachers and infrastructure, and balanced development among schools. Especially in mathematics education, mathematics teachers often have more unique teaching art and teaching strategies. For example, they often create a series of mathematical problem situations to stimulate students’ cognition, so that students can understand the whole process of mathematical problem-solving (Zang, 2006). Thus, the students’ overall mathematics academic level and mathematical problem-solving ability are relatively good, and students are especially able to successfully deal with mathematics problems of medium and high difficulty levels. Nevertheless, it cannot be overlooked that this study mainly relies on paper-and-pencil tests for the monitoring of mathematics problem-solving, and the East Asian middle school students participating in the PISA survey may have faced a more complex problem-solving test environment (the testing process, for instance, relied on computer technology); thus, their problem-solving ability performance may have been easily underestimated.

### Differences in the benchmark of middle school students’ mathematical problem-solving ability in mainland China

In this study, significant differences are observed with regard to gender and only-child situation in terms of mathematical problem-solving benchmark among students from different backgrounds, these differences are not practical. Many studies have also pointed out that no statistical difference exists in students’ mathematical ability based on gender (Fennema and Sherman, 1976). However, from the perspective of cultural tradition, men in East Asia tend to have more educational expectations than women, which interferes with academic performance and mathematical ability (Zhu et al., 2018). On the other hand, retrospecting China’s population policy changing, the sex ratio of the domestic population decreased from 107.56 in 1953 to 104.88 in 2021. Moreover, from the development trend, although it has been declining, the total number of men is still higher than that of women, and it is worth noting that the gender ratio of the population in East China is also higher than the national average (Yuan and Wu, 2022). Overall，Chinese boys are more likely than girls to perform at higher levels in problem-solving. As for family structure, according to the resource dilution theory, only children who receive family support are more likely to succeed in academic performance and mathematical ability improvement (Blake, 1981). In contrast, the differences in the performance of students’ mathematical problem-solving abilities caused by different urban and rural backgrounds have more practical significance, this may be due to the significant, long-term urban–rural education gap in mainland China. In reality, although the country vigorously implements the policy of *Coordinated Development of Compulsory Education in Urban and Rural Areas*, objectively, the situation of urban education resources concentration and urban family education investment surge has not reversed, so the current situation of relatively weak education quality in districts, counties, towns and rural schools cannot be changed in the short term (Liu, 2006; Wei, 2018). Moreover, even in East China, where the development of basic education is relatively balanced, the educational differences between urban and rural areas are still significant. But the difference is that the gap between urban and rural education in East China is more about the quality of teachers than the hardware conditions such as infrastructure. For example, urban teachers can often get more high-level education and training opportunities (including the interpretation of mathematics curriculum standards), so they have a more accurate grasp of many teaching contents and more effective teaching methods (Zang, 2006). Therefore, on the whole, the performance of mathematical problem solving ability of urban students in Z Province is better than that of students in counties, towns and rural areas.

### The predictive effect of student-level and school-level factors on mathematical problem-solving ability

On the whole, this study points out that 28% of the difference in mathematical problem-solving performance among middle school students in East China comes from inter-school variation, which shows that the imbalance of problem-solving between schools in compulsory education in mainland China still requires attention. According to the analysis results of the inter-school differences in PISA 2012, the percentage of the average variation in mathematical problem-solving performance among OECD members, accounting for school characteristics, is 38%. Simultaneously, the percentage of Shanghai samples who participated in the test on behalf of mainland China reaches 42%, while the mathematical problem-solving performance of students in countries such as Finland and Sweden is relatively balanced, with an average variation in problem-solving results across schools lower than 20% (OECD, 2014). The above results fully indicate that there is still space for improvement in the inter-school differences in the mathematical problem-solving of students in compulsory education in mainland China. As far as the current education situation in East China is concerned, the overall development level of basic education is relatively balanced, so the inter school differences in students’ mathematical performance are not particularly prominent, which is mainly due to the positive measures taken in this region, such as paying attention to education layout planning and increasing support for weak schools (Zang, 2006). However, due to the long-term existence of urban–rural dual economic and social development structure, local weak rural schools have always been at a disadvantage in solving problems, and their school running quality and education investment are obviously insufficient (Liu, 2006). In addition, under the influence of social class differentiation, the average socioeconomic status of schools composed of students with different family socioeconomic statuses further exacerbates the Matthew effect of inter-school differences in mathematical problem-solving (Dumay and Dupriez, 2008).

In addition to the significant difference in the performance of middle school students’ mathematical problem-solving ability caused by the gap between urban and rural backgrounds, the study also found that students’ individual non-intelligence factors (e.g., mathematics interest, self-efficacy, teacher–student relationship, and mathematics anxiety) explained the difference in mathematical problem-solving more than students’ background variables (e.g., gender, only-child situation, and leftover situation) did. The development of individual characteristics is always accompanied by the psychological maturity of students, which, when compared to individual background, may better predict problem-solving (Lu, 2011; Alibali et al., 2019). In addition, some studies have found that increasing middle school students’ mathematics interest and self-efficacy can effectively improve their mathematical problem-solving, while excessive mathematics anxiety can hinder it (Xu and Qi, 2018). Similarly, this study demonstrated that mathematics interest, self-efficacy, and teacher–student relationship positively influenced students’ problem-solving, while mathematics anxiety negatively affected it. This is because positive learning attitudes and persistence can promote mathematical thinking, while poor learning attitudes and habits can hinder mathematics learning and thinking (Huang, 2006). In view of this, in the future, mathematics teaching of secondary schools in various countries should pay more attention to the regulating role of non-intellectual factors like mathematical affections in the process of problem solving, such as actively creating mathematical problem situations to promote their interest in mathematics learning, increasing the opportunities for students about problem posing, and alleviating the anxiety of mathematical problem solving.

Furthermore, this study remarks that teaching factors are important for students’ mathematical problem-solving ability; in particular, the teaching methods of mathematical problem-solving have played an important role in nurturing this ability because real teaching scenarios can provide students with step-by-step decomposition and reasoning analysis of the problem-solving process (Pasani, 2018). Therefore, for the cultivation of mathematical problem-solving ability in middle schools, the primary task of future mathematical classroom teaching is to improve the teaching strategy of problem-solving to activate students’ mathematical cognition, such as appropriately transforming some open-ended problems with complex problem situations to help students gradually develop their mathematical thinking in the process of exploring the procedures of problem solving.

### Limitations

This research has some limitations. First, although the *Regional Educational Quality and Health Monitoring* project adopted a relatively scientific PPS sampling method and included school students from different districts and counties and urban and rural backgrounds, such as administrative divisions, the main source was a sample of students from the upper levels of education and economy in East China. Therefore, the main findings of this study can provide appropriate reference for mathematics education in the developed regions of other countries, but at the same time, some conclusions still cannot be extended to the other regions of mainland China. For example, there may be differences between leftover and non-leftover students in China’s underdeveloped provinces in mathematical problem-solving performance. In view of this, the follow-up research can further enrich the survey samples, such as expanding to the whole country. Second, from the type of math problem solving in test paper, the authors mainly use the two forms of multiple-choice questions and subjective questions commonly used in math tests in mainland China, which have less reference for the problem solving test questions in mathematics textbooks for secondary schools from other countries. Therefore, future research can consider from the perspective of textbook analysis to further enrich the form of math problem presentation in the current test paper, so as to facilitate subsequent international comparisons. Third, due to the limitation of the variables in the database, as this study did not choose SES and the school average SES as the optimal control variables in different levels but instead replaced them with the leftover situation and urban or rural background situation, the estimated results present deviations to a certain extent. Finally, limited by the volume of the questionnaire survey, the factors affecting the mathematical problem-solving ability selected in this study only involved students’ background and internal non-intellectual factors, with less consideration of factors such as meta-cognition, including learning strategies, which may lead to limitations in the process of impact mechanism analysis. Thus, follow-up supplementary research could consider increasing the content of the student questionnaire on the influencing factors of mathematical problem-solving ability.

## Conclusion

This study focused on the systematic monitoring and investigation of mathematical problem-solving ability of middle school students in the compulsory education stage in mainland China. It addressed the overall proficiency, background differences in the benchmark of ability, and the predictive effect of student-level and school-level factors on mathematical problem-solving performance, drawing meaningful conclusions.

First, the mathematics problem-solving ability of middle school students in Z province in mainland China is relatively good, and 96% of the students’ mathematics problem-solving ability meets the basic academic requirements of the curriculum standards.

Second, in the difference analysis of the benchmark for middle school students’ mathematical problem-solving ability performance, we found that the proportion of boys reaching the C level and above was significantly higher than that of girls, and the proportion of only children reaching the C level and above was also significantly higher than that of non-only children. In contrast, the proportion of non-leftover students reaching the C level and above was higher than that of leftover students, but no significant difference was observed between the two.

Finally, in terms of school-level variables, urban and rural backgrounds had a larger impact on mathematical problem-solving than teaching factors. Among the teaching factors, the teaching method of problem-solving had a relatively greater positive impact on problem-solving than the teaching self-efficacy. For student-level variables, the influence of individual non-intellectual factors on mathematical problem-solving was higher than that of student background variables, including a greater positive effect of self-efficacy and a higher negative effect of mathematics anxiety. Moreover, among the effects of student background on mathematical problem-solving, gender had the largest negative effect, followed by the effect of the only-child situation, while the positive impact of the leftover situation was not significant. In particular, only children and boys performed better.

## Author’s note

The samples used in this study were provided by the *Regional Educational Quality and Health Monitoring* project. The schools and education bureaus in the participating areas signed cooperative research agreements with the project team.

## Data availability statement

The datasets presented in this article are not readily available because in view of the data confidentiality agreement, the data used in this study is only shared by members of the project team and cooperative institutes. Requests to access the datasets should be directed to qichuxia@126.com.

## Ethics statement

The studies involving human participants were reviewed and approved by the Academic Committee of the Faculty of Education in Beijing Normal University. Written informed consent to participate in this study was provided by the participants’ legal guardian/next of kin.

## Author contributions

ZX wrote this manuscript. CQ data provided and theoretical guidance. All authors contributed to the article and approved the submitted version.

## Funding

This study was funded by General Program of NSFC: “Research on Cognition and Brain Mechanism of Students’ Mathematical Creative Thinking in Complex Situations (no: 62277003)” and the project of The China Basic Education Quality Monitoring Collaborative Innovation Center: “Middle School Mathematics Literacy Evaluation and Diagnostic Improvement (no: 110105006)”.

## Conflict of interest

The authors declare that the research was conducted in the absence of any commercial or financial relationships that could be construed as a potential conflict of interest.

## Publisher’s note

All claims expressed in this article are solely those of the authors and do not necessarily represent those of their affiliated organizations, or those of the publisher, the editors and the reviewers. Any product that may be evaluated in this article, or claim that may be made by its manufacturer, is not guaranteed or endorsed by the publisher.

## Footnotes

^1^*φ* represents the effect size of the Chi-squared test.

^2^The Angoff method is one of the most commonly used in standard setting procedures and could be also used to determine the academic benchmark. Specifically, two or more split points were used in large-scale assessments to classify students’ academic performance into multiple levels to determine classification criteria for different proficiency.

^3^In this study, the performance of the influencing factors is represented by the average value of multiple items that affect students’ mathematical problem-solving ability.
